# Selectively enhanced photocurrent generation in twisted bilayer graphene with van Hove singularity

**DOI:** 10.1038/ncomms10699

**Published:** 2016-03-07

**Authors:** Jianbo Yin, Huan Wang, Han Peng, Zhenjun Tan, Lei Liao, Li Lin, Xiao Sun, Ai Leen Koh, Yulin Chen, Hailin Peng, Zhongfan Liu

**Affiliations:** 1 Center for Nanochemistry, Beijing Science and Engineering Center for Nanocarbons, Beijing National Laboratory for Molecular Sciences, College of Chemistry and Molecular Engineering, Peking University, 202 Chengfu Road, Haidian District, Beijing 100871, China; 2 Clarendon Laboratory, Department of Physics, University of Oxford, Parks Road, Oxford OX1 3PU, UK; 3 Academy for Advanced Interdisciplinary Studies, Peking University, Beijing 100871, China; 4 Stanford Nano Shared Facilities, Stanford University, Stanford, California 94305, USA

## Abstract

Graphene with ultra-high carrier mobility and ultra-short photoresponse time has shown remarkable potential in ultrafast photodetection. However, the broad and weak optical absorption (∼2.3%) of monolayer graphene hinders its practical application in photodetectors with high responsivity and selectivity. Here we demonstrate that twisted bilayer graphene, a stack of two graphene monolayers with an interlayer twist angle, exhibits a strong light–matter interaction and selectively enhanced photocurrent generation. Such enhancement is attributed to the emergence of unique twist-angle-dependent van Hove singularities, which are directly revealed by spatially resolved angle-resolved photoemission spectroscopy. When the energy interval between the van Hove singularities of the conduction and valance bands matches the energy of incident photons, the photocurrent generated can be significantly enhanced (up to ∼80 times with the integration of plasmonic structures in our devices). These results provide valuable insight for designing graphene photodetectors with enhanced sensitivity for variable wavelength.

## Abstract (web summary)

Graphene has the high carrier mobility and short photoresponse time required for efficient photodetection, but broad and weak optical absorption are severe drawbacks. Here, the authors show that twisted bilayer graphene with van Hove singularities exhibits a strong light-matter interaction and selectively enhanced photocurrent generation.

## Graphical abstract

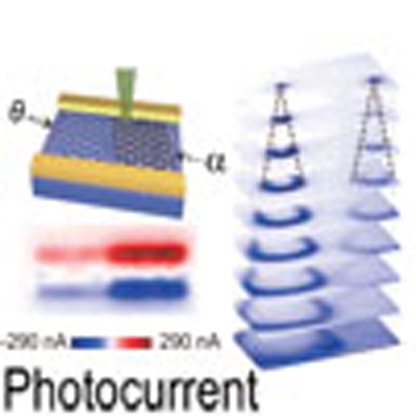

The unique Dirac-cone band structure makes graphene a promising material for photodetection. Its linearly dispersive band structure near Fermi level results in massless Dirac fermion type of carriers, large Fermi velocity (∼1/300 of the speed of light) and surprisingly high carrier mobility^1^,^2^,^3^,^4^. In graphene device, the photovoltage generation time is shorter than 50 fs, which is associated with the carrier heating time^5^. In addition, the rapid cooling process of photoexcited carriers (∼picoseconds) in the monolayer graphene results in a quick annihilation of photoelectrical signal in the electric circuit^5^,^6^,^7^,^8^,^9^,^10^,^11^,^12^. These advantages of the monolayer graphene facilitate its applications associated with ultrafast photodetection, such as high-speed optical communications^13^,^14^,^15^,^16^,^17^ and terahertz oscillators^18^. However, it remains a great challenge to achieve high photoresponsivity and selectivity in the monolayer-graphene-based detectors due to the weak and broadband absorption (only 2.3%, from the ultraviolet to the infrared)^19^ and the short photocarrier cooling time (∼picoseconds)^5^,^6^,^7^,^8^,^9^,^10^,^11^,^12^.

On the other hand, twisted bilayer graphene (tBLG) is non-AB stacked bilayer graphene in which one graphene monolayer sheet rotates by a certain angle (*θ*) relative to the other (Fig. 1a). Recent theoretical studies of tBLG have shown that the Dirac band dispersions change dramatically and become strongly warped with small twist angles (*θ*≤5°)^20^,^21^,^22^,^23^. Even at relatively large twist angles, the electronic coupling between the two monolayers, albeit weak, can still introduce new band structures^20^,^21^,^22^,^23^,^24^,^25^,^26^,^27^. Unlike the parabolic band structure in AB-stacked bilayer graphene^28^,^29^,^30^,^31^,^32^,^33^,^34^, the band structure of tBLG with large twist angle (typically larger than 5°)^20^,^35^ maintains linear near the Dirac point and thus it inherits some unique properties of monolayer graphene^36^. Away from the Dirac point, Dirac cones of the two individual monolayers intersect and form saddle points in reciprocal space of tBLG^24^, leading to the formation of van Hove singularities (VHSs) in the density of state (DOS)^25^,^26^,^35^,^37^,^38^, which then gives rise to some interesting phenomena such as enhanced optical absorption, Raman G-band resonance and enhanced chemical reactivity of tBLG^27^,^37^,^39^,^40^,^41^,^42^,^43^,^44^,^45^.

In this study, to address the problem of low photoresponsivity and selectivity in the monolayer graphene photodetection, we explore the high-performance photodetector based on tBLG with VHSs. For the first time, we report that the VHSs in tBLG leads to a prominent photocurrent enhancement of tBLG photodetectors with a wavelength selectivity under incident light irradiation.

## Results

### Structure and Raman spectra

**Figure 1:**
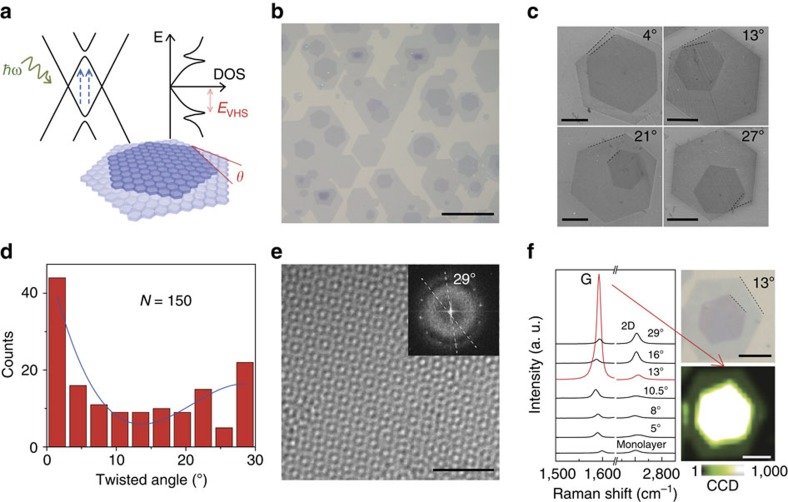
Structures and Raman spectra of tBLG with different twist angles. (**a**) Schematics for band structure with minigaps (top left) and the corresponding DOS with VHSs (top right) in tBLG (bottom). Blue arrows describe the photoexcitation process as the energy interval of two VHSs (2*E*_VHS_) matches the energy of incident photon. (**b**) The optical image of tBLG domains grown by CVD on Cu and then transferred onto SiO_2_ (90 nm)/Si substrate. Scale bar, 30 μm. (**c**) Scanning electron microscopy (SEM) images of tBLG domains with different twist angles on SiO_2_/Si. The twist angles are measured from the edges of over- and underlayer of tBLG domains. Scale bars, 5 μm. (**d**) Histogram of twist angles measured from tBLG domains in the CVD sample as shown in **b**. (**e**) Typical high-resolution TEM (HRTEM) image of tBLG. The periodicity of the moiré pattern is ∼0.455 nm. The inset is the fast Fourier transform (FFT) of the image, showing that the twist angle is 29°. Scale bar, 2 μm. (**f**) Left column, Raman spectra of monolayer graphene and tBLG domains with twist angle of 5°, 8°, 10.5°, 13°, 16° and 29°, respectively. The incident laser wavelength is 532 nm (2.33 eV). Top right: the optical image of 13° tBLG domain on SiO_2_/Si. Bottom right: G-band intensity mapping image of the 13° tBLG domain shows uniformity of the intensity enhancement of Raman G-band. Scale bars, 10 μm.

tBLG samples were grown on copper foil via chemical vapour deposition (CVD) method and then transferred to heavily doped Si substrate, which was capped with 90 nm SiO_2_. As shown in typical optical image and scanning electron microscopy images (Fig. 1b,c), both the overlayer and underlayer in tBLG exhibit hexagonal shapes with sharp edges, which implies highly crystalline qualities of tBLG domains^46^,^47^,^48^. The interlayer twist angle can be measured from the relative misalignment of the straight edges, which is consistent with the observation by transmission electron microscopy (TEM) (Supplementary Fig. 1 and Supplementary Note 1). tBLG domains with different twist angles can be readily obtained in our samples (Fig. 1d), which provide a platform for the study of *θ*-dependent light–matter interactions. The highly crystalline quality and clean interface between two monolayers of our CVD sample are evidenced by the moiré pattern in high-resolution TEM image (Fig. 1e). This clean interface guarantees the interaction and coupling of electronic states from the over- and underlayer of tBLG. This interlayer electronic coupling is also proved by the enhanced G-band peak in Raman spectra (Fig. 1f). Taking 13° tBLG domain as an example, the Raman G-band intensity displays a tremendous enhancement of ∼20 folds under 532 nm laser (2.33 eV), which is consistent with the previously reported results^27^,^28^,^39^,^40^,^41^,^42^,^43^. This Raman G-band enhancement implies that an interlayer coupling introduces new band structures in tBLG. In addition, the enhanced G-band intensity of 13° tBLG domain was found to be uniform across the whole domain as shown in the G-band mapping image (Fig. 1f), which further confirms the high quality of our CVD tBLG samples. The Raman G-band enhancement is believed to correlate with the formation of VHSs in tBLG^27^,^37^,^39^,^40^,^41^,^42^,^49^.

### Micro-ARPES spectra of tBLG

**Figure 2:**
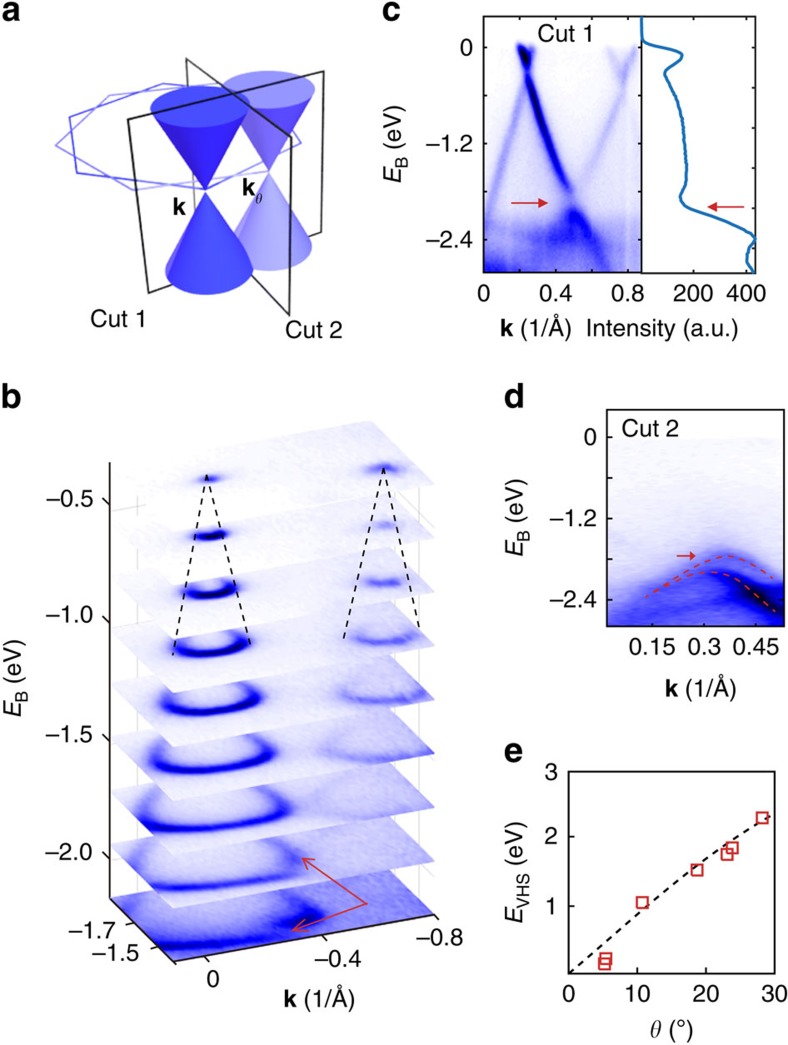
Micro-ARPES spectra of tBLG. (**a**) Schematic illustration of the first primitive Brillouin zones (hexagons) and Dirac cones of over- and underlayer of tBLG. (**b**) Stacking plot of constant-energy contours at different binding energies (*E*_B_) of tBLG. (**c**) ARPES spectra along Cut 1 as labelled in **a**. The right curve is energy spectrum density curve (EDC) integrated from the spectrum. (**d**) ARPES spectra along Cut 2 as labelled in **a**. Red arrows in **b**,**c** and **d** indicate the minigap band topology and the split parallel branches arising from interlayer coupling. (**e**) *E*_VHS_ versus the twist angle (*θ*) of tBLG domains. The *E*_VHS_, measured from micro-ARPES data of tBLG, is the energy interval between the minigap (VHS) and Dirac point. The *E*_VHS_ varies almost linearly with twist angle. The black dashed line is a theoretical curve.

To unravel the nature of VHSs, we directly investigate the band structures of CVD-grown tBLG domains using spatially resolved angle-resolved photoemission spectroscopy with submicrometre spatial resolution (micro-ARPES). Owing to the twist angle (*θ*) between over- and underlayer of tBLG, the two sets of (six) Dirac points originated from each layer are rotated relatively by the angle *θ* as well (see Fig. 2a), which we mark as the **k** (left cones) and **k**_*θ*_ (right cones) points, respectively. The band structures of a tBLG domain are shown in Fig. 2, where the constant energy contours (Fig. 2b), the band dispersions cutting across (Fig. 2c) and perpendicular (Fig. 2d) to the two adjacent Dirac points are presented, respectively. In Fig. 2b, the stacking plots of the band contours at different binding energies clearly depict the typical two Dirac-cone dispersions of tBLG and each preserves the linear dispersion of monolayer graphene. One of the Dirac cone exhibits a weaker intensity and higher electron doping level, indicating its origin from the underlayer graphene, as the photoelectrons from the bottom layer are screened by the top layer (thus leading to a weaker intensity), and being closer to the Cu substrate also increases its charge transfer^50^,^51^.

By measuring the separation between the two Dirac points (Fig. 2b,c), we can determine the twist angle (*θ*) of this tBLG domain as 19.1° (Supplementary Fig. 2 and Supplementary Note 2). Without interlayer coupling, the two Dirac cones in Fig. 2 shall intersect and cross each other at higher binding energy. Instead, the band structure at Fig. 2b clearly shows fine structures at the intersection (indicated by red arrows in Fig. 2b) and the dispersion in Fig. 2c shows the opening of the gap at the crossing point of the dispersions from the two Dirac cones, which is indicated by the faint intensity in the spectra intensity map (left panel) and the dip in the DOS plot (right panel, indicated by red arrows). This gap opening in the band structure is a typical anticrossing behaviour introduced by interlayer electronic coupling^24^, which leads to the formation of the VHS (Fig. 1a). In addition, from Fig. 2d, one can see that the anticrossing affects the hyperbolic curve as well and results in split and parallel dispersions.

With the same method, we further studied tBLG domains with various different twist angles and tracked the positions of VHSs with respect to the twist angles, as can be seen in Fig. 2e. At small angles, the value of *E*_VHS_ increases almost linearly with *θ*, in consistence with the theoretical prediction (Supplementary Fig. 2). This dependence also helps explain the Raman G-band enhancement at specific twist angle (Fig. 1f) for a given incident laser frequency. If the energy of incident photon matches the energy interval of the two VHSs of tBLG (*ℏω*≈2*E*_VHS_, see Fig. 1a), the electrons are excited and transit between the fine band structure, causing the increase of the intensity of Raman G band peak (see Supplementary Fig. 3 and Supplementary Note 3 for details).

### Selectively enhanced photocurrent generation of tBLG

**Figure 3:**
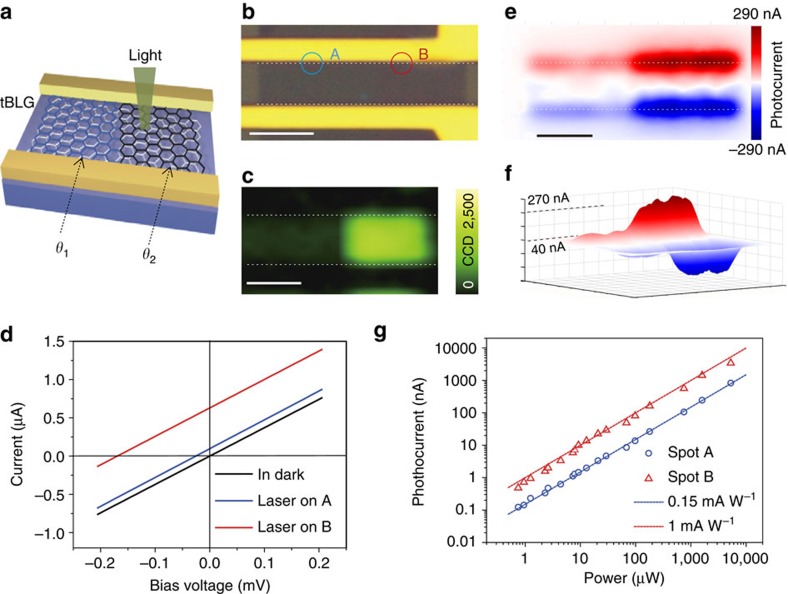
Selectively enhanced photocurrent generation in tBLG photodetection devices. (**a**) Schematic illustration of a tBLG photodetection device. The channel comprises of two adjacent tBLG domains with different twist angles of *θ*_1_ and *θ*_2_, respectively. (**b**) Optical image of the tBLG photodetection device. The *θ*_1_ and *θ*_2_ are 7° and 13°, respectively. (**c**) Raman G-band intensity mapping image under 532 nm (2.33 eV) laser. 13° tBLG domain exhibits an enhanced G-band intensity. (**d**) Current versus source-drain bias (*I*–*V*) curve without laser on and with laser focusing on 7° (spot A) and 13° (spot B) tBLG domains, respectively. The intercepts at current axis represent the net photocurrents. (**e**) Scanning photocurrent images of the same tBLG device. A 532-nm laser with power of 200 μW is focused on the device, while the net photocurrent is amplified and then detected by a lock-in amplifier. All the photocurrents here are generated without source-drain bias and gate bias. (**f**) Three-dimensional view of the scanning photocurrent image of the same tBLG device. (**g**) Photocurrents generated from 7° (spot A) and 13° (spot B) tBLG domains as a function of incident power, respectively. The white dashed lines in **c** and **e** show the positions of graphene–metal electrode interfaces, respectively. Scale bars, 5 μm (all).

The strong light–matter interaction of tBLG selectively enhanced by the VHSs can also enhance the generation of photocurrent under illumination. As an example, two adjacent tBLG domains with twist angle of 13° and 7° transferred onto SiO_2_ (90 nm)/Si were etched into a strip and then embedded into two-terminal devices in parallel (Fig. 3a,b). Raman spectroscopy and two-dimensional maps of the two adjacent tBLG domains were first measured under the 532-nm laser (2.33 eV). As expected, the G-band intensity of whole 13° domain exhibits a uniformly 20-fold enhancement as compared with the 7° domain (see Fig. 3c), as the energy interval of the two VHSs in 13° domain matches the energy of incident photon (*ℏω*≈2*E*_VHS_). To generate photocurrent selectively, interfacial junctions of tBLG-metal electrodes were used to separate the photoexcited electrons and holes under illumination^52^,^53^. As shown in current–bias voltage curves, both tBLG domains produce pronounced photocurrent shifts (Fig. 3d). Remarkably, the 13° tBLG domain generates a much larger net photocurrent (0.63 μA) at zero bias than that of the 7° domain (0.097 μA), originated from selectively enhanced light–matter interaction of 13° tBLG domain with the 532-nm laser.

We further conducted net photocurrent mapping of the device by using scanning photocurrent microscopy, in which the net photocurrent was recorded while scanning a focused 532-nm laser spot with a diameter of ∼1 μm over the device (Fig. 3e,f). The photocurrent was observed to exhibit contrary directions at the two graphene–metal electrode interfaces in the device. Significantly, the intensity of photocurrent generated at 13° tBLG domain is ∼6.6 times stronger than that at the 7° tBLG domain. This twist angle-related photocurrent enhancement holds great promise in high-selectivity photodetection applications.

To further evaluate the photoresponsivity of tBLG, we performed photocurrent measurements of tBLG devices under different incident power of 532 nm laser illumination, respectively. As shown in Fig. 3g, the photocurrents from 7° and 13° tBLG domains both increase as the incident power rises from ∼1 μW to ∼5 mW. The photoresponsivity of 7° and 13° tBLG domain is measured as ∼0.15 and ∼1 mA W^−1^, respectively, indicating a robust and strong enhancement in 13° tBLG domain under different incident power of 532 nm laser illumination.

**Figure 4:**
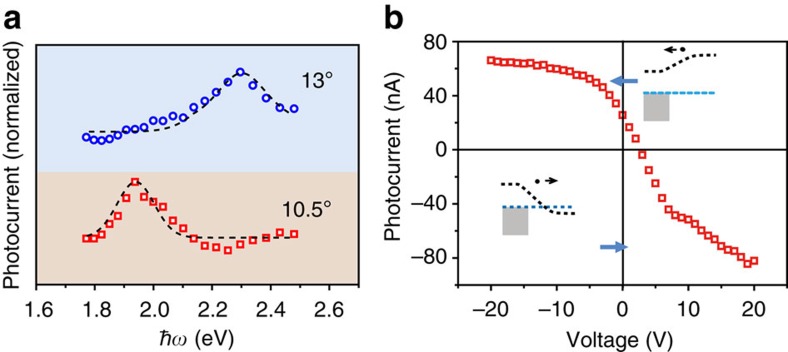
The variation of photocurrent with photon energy and gate voltage. (**a**) Photocurrent versus energy of incident photon (*ℏω*). tBLG domains with 10.5° and 13° twist angles show different peak positions. Incident photons with energy *ω* near 2*E*_VHS_ generate an enhanced photocurrent, while photons with energy lower or higher than 2*E*_VHS_ excite ordinary optoelectronic processes. Dotted lines were used to guide the eyes. The plots are normalized with that of AB-stacked bilayer graphene. (**b**) Plot of photocurrent as function of gate voltage. Insets are the corresponding band profiles, where the grey boxes, blue dotted lines and black dotted lines represent Ti electrodes, Fermi levels and positions of Dirac points of tBLG, respectively.

From the unravelling of band structures, the energy interval of the two VHSs (2*E*_VHS_) of 13° tBLG domain is ∼2.34 eV, which matches the energy of incident photon (2.33 eV, *λ*=532 nm) and thus leads to a strong light–matter interaction. When we changed the wavelength of incident laser from 532 to 632.8 nm (1.96 eV), the photocurrent was found to be selectively enhanced in a 10.5° tBLG domain device with 2*E*_VHS_ of ∼1.89 eV (Supplementary Fig. 4 and Supplementary Note 4). To further investigate the correlation of 2*E*_VHS_ with *ℏω* in photocurrent generation of 13° and 10.5° tBLG domains, *ℏω* was gradually changed from 1.77 to 2.48 eV (500 to 700 nm in wavelength), while the power of incident laser was kept unchanged. As shown in Fig. 4a, the photocurrents of 13° and 10.5° tBLG domains exhibit peaks at ∼2.30 and ∼1.94 eV, agreeing well with 2*E*_VHS_ values (∼2.34 and ∼1.89 eV), respectively.

## Discussion

The origin of the photocurrent enhancement can be understood qualitatively when taking the unique electronic state of tBLG into account. In the photoexcitation process, the interband transition has to satisfy both momentum and energy conservation. For momentum conservation, the electrons are confined to transit between states with the same **k** value in reciprocal space, owing to the very small momentum of incident photons. As for energy conservation, the energy difference between these two states equals to *ℏω*. When *ℏω≈*2*E*_VHS_, the initial and final states are both near VHSs (thus with enhanced DOS, see Fig. 1a). Specifically, the effect of VHSs on the photoexcitation process can be evaluated by joint DOS (JDOS), which is defined as:


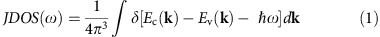


where *E*_C_ and *E*_V_ represent the energies of the conduction and valence bands, respectively. JDOS is associated with the process in which an electron absorbs a photon with energy *ℏω*=*E*_c_−*E*_v_ and then transits from conduction to valence band. A calculated JDOS shows an abrupt increase associated with the VHSs when *ℏω≈*2*E*_VHS_ (ref. 40). This leads to an enhanced photoexcitation process, consistent with experimental observations in Raman (Fig. 1f) and absorption spectra^27^,^45^. As a result, the intensified photoexcitation process may result in the enhanced photocurrent generation.

Besides the efficient photoexcitation, the improvement of separation efficiency of excited carriers can facilitate the photocurrent generation in tBLG. A gate voltage applied on the tBLG photodetection device can manipulate the doping level of graphene in channel and thus change the value of Seebeck coefficient, which may simultaneously lead to the photocurrent change^54^,^55^,^56^,^57^. As shown in Fig. 4b, the photocurrent of 13° tBLG domain has a 2.6-fold increase from ∼25 to ∼66 nA when the back-gate voltage decreases from 0 to −20 V. As the back-gate voltage increases from 0 to 20 V, the photocurrent first flips its polarity at 2.5 V and then reaches a value of about −82 nA. The inset in Fig. 4b shows the two band profiles of graphene–metal electrode junctions in the tBLG photodetection device under the applied back-gate voltage. From the transfer curve (Supplementary Fig. 5 and Supplementary Note 5), we believe that the positive gate voltage manipulates the graphene in channel from *p*- to *n*-type doping, which gives rise to the change of Seebeck coefficient. In contrast, owing to the Fermi-level pinning of graphene underneath the metal electrodes, its Seebeck coefficient keeps unchanged. Therefore, the difference of these two Seebeck coefficients could be tuned and flipped by gate voltage, which leads to the value and polarity change of photocurrent.

**Figure 5:**
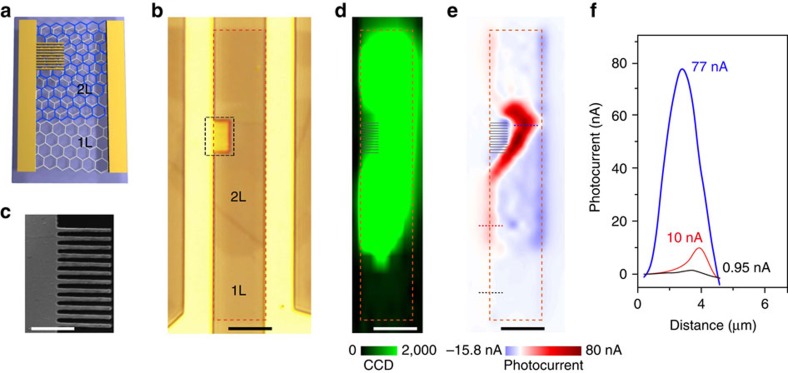
tBLG photodetector integrated with plasmonic structure. (**a**) Schematic illustration of the detector. The channel comprises graphene monolayer domain and tBLG domain which are labelled by ‘1L' and ‘2L', respectively. The electrode is integrated with finger structure. (**b**) Optical image of the tBLG photodetector. (**c**) Scanning electron microscopy (SEM) image of the finger structure labelled by black dashed rectangle in **b**. (**d**) Raman G-band intensity mapping image of the device under 532 nm laser. The tBLG domain exhibits an enhanced G-band intensity. (**e**) Scanning photocurrent image of the photodetector. The exciting light is a focused 532 nm laser with power of 30 μW. (**f**) Line-scanning photocurrent of the photodetector. The blue, red and black curves correspond to photocurrent distributions along the tBLG near figure structure, tBLG and graphene monolayer, which are labelled by blue, red and black dashed lines in **e**. The maximum photocurrent at tBLG with the plasmonic structure is 77 nA, while the maximum photocurrents at the tBLG and monolayer graphene are 10 and 0.95 nA, respectively. All the photocurrents here are generated without source-drain bias and gate bias. The red dashed rectangles in **b**,**d** and **e** correspond to the channel of the photodetector. Scale bar, 1 μm (finger structure in **c**). Scale bars, 5 μm (others).

The responsivity of tBLG is measured as ∼1 mA W^−1^ at the resonance frequency, which is about 20 times enhancement compared with that of mechanically exfoliated monolayer graphene (∼0.05 mA W^−1^) with similar device configuration (Supplementary Fig. 6). To further improve the responsivity, we have integrated tBLG with plasmonic electrode structures as shown in Fig. 5a. A tBLG domain and an adjacent monolayer domain were embedded into the same two-terminal electrodes. A finger-patterned plasmonic structure (Ti/Au, 5/45 nm in thickness) with 110 nm finger width and 300 nm pitch^58^ were fabricated on the tBLG domain as shown in Fig. 5b,c. The Raman mapping image in Fig. 5d exhibits uniformly enhanced G-band intensity, which confirms that the interval of two VHSs (2*E*_VHS_) of the tBLG domain matches the energy of incident photon (532 nm and 2.33 eV). The scanning photocurrent results of the device (Fig. 5e,f) show that the photocurrents of tBLG and the adjacent monolayer domain are measured as ∼10 and ∼0.95 nA, respectively. The enhancement of about 10.5 times is achieved. Remarkably, the photocurrent generated on tBLG near the finger structure is further enhanced to the value of ∼77 nA, which is ∼80-fold enhancement compared with the photocurrent of adjacent monolayer graphene channel. The mechanism of such strong photoresponsivity enhancements can be ascribed to the combination of the selective resonance enhancement of VHSs in tBLG and plasmonic enhancement of the finger structure.

In summary, we have experimentally demonstrated that the light–matter interaction in tBLG with VHSs is dependent on the interlayer twist angle (*θ*) and then can lead to a selective enhancement in photocurrent generation. Micro-ARPES was performed to unravel the band structure of CVD-grown tBLG and reveal the emergence of *θ*-dependent VHSs. The photocurrent of tBLG photodetectors exhibits a ∼6.6-fold enhancement at a suitable *θ*, when the energy interval of the VHSs (2*E*_VHS_) matches the energy of incident photon (*ℏω*). By integrating plasmonic structures, the responsivity of tBLG photodetector can be significantly enhanced by ∼80 folds compared with the monolayer graphene. Our results open a new route to graphene-based optoelectronic applications.

## Methods

### tBLG growth and characterization

The tBLG was grown on copper foil in a home-made low-pressure CVD system. The growth was carried out under the flow of H_2_ and CH_4_ (600:1 in volume) with a pressure of 600 Pa at 1,030 °C for 40 min. The samples were characterized by Olympus BX51 microscope, scanning electron microscopy (Hitachi S-4800 operated at 2 kV), TEM (FEI Tecnai F30 operated at 300 kV for the diffraction image and FEI 80-300 Cs image-corrected Titan operated at 80 kV for the moiré pattern image) and Raman spectroscopy (Horiba HR800). The micro-ARPES measurements were carried out at the spectromicroscopy beamline at Elettra Synchrotron Radiation lab in Italy, with energy resolution of 50 meV, spatial resolution of 0.8 μm and angle resolution of 0.5°. Before the micro-ARPES measurements, *in-situ* annealing at 350 °C was carried out to clean the sample surface.

### Device fabrication and measurement

tBLG samples on copper were transferred to a highly doped Si substrate with 90 nm SiO_2_ with the help of poly(methyl methacrylate)^59^. The Ti/Au (20/30 nm) electrodes and Ti/Au (5/45 nm) figure structures were fabricated by electron-beam lithography and the following electron-beam evaporation. The electrical measurements were performed by Keithley SCS-4200. The photoelectrical measurements were performed by a scanning photocurrent microscopy. In the set-up, 532 and 632.8 nm, and Supercontinuum Laser Sources (NKT Photonic) were used as laser sources. The chopper-modulated (∼1 kHz) laser beams were focused to ∼1 μm on the device using × 100 objective and the short-circuit photocurrents were then measured by pre-amplifier and lock-in amplifier. When scanning the laser spot over the device, the induced photocurrents and beam positions were recorded and displayed simultaneously with the assistance of a computer, which communicated with lock-in amplifier and motorized stage (with device on it). A voltage source (Keithley 2400) was used to supply the gate voltage. All the electrical and photoelectrical measurements were performed in air at room temperature.

## Additional information

**How to cite this article:** Yin, J. *et al.* Selectively enhanced photocurrent generation in twisted bilayer graphene with van Hove singularity. *Nat. Commun.* 7:10699 doi: 10.1038/ncomms10699 (2016).

## Supplementary Material

Supplementary Information Supplementary Figures 1-6, Supplementary Notes 1-5 and Supplementary References.

## References

[b1] Novoselov K. S. *et al.* Two-dimensional gas of massless Dirac fermions in graphene. Nature 438, 197–200 (2005). 16281030 10.1038/nature04233

[b2] Novoselov K. S. *et al.* Electric field effect in atomically thin carbon films. Science 306, 666–669 (2004). 15499015 10.1126/science.1102896

[b3] Zhang Y. B. *et al.* Experimental observation of the quantum Hall effect and Berry's phase in graphene. Nature 438, 201–204 (2005). 16281031 10.1038/nature04235

[b4] Geim A. K. & Novoselov K. S. The rise of graphene. Nat. Mater. 6, 183–191 (2007). 17330084 10.1038/nmat1849

[b5] Tielrooij K. J. *et al.* Generation of photovoltage in graphene on a femtosecond timescale through efficient carrier heating. Nat. Nanotechnol. 10, 437–443 (2015). 25867941 10.1038/nnano.2015.54

[b6] Sun D. *et al.* Ultrafast relaxation of excited Dirac fermions in epitaxial graphene using optical differential transmission spectroscopy. Phys. Rev. Lett. 101, 157402 (2008). 18999638 10.1103/PhysRevLett.101.157402

[b7] George P. A. *et al.* Ultrafast optical-pump terahertz-probe spectroscopy of the carrier relaxation and recombination dynamics in epitaxial graphene. Nano Lett. 8, 4248–4251 (2008). 19367881 10.1021/nl8019399

[b8] Tani S. *et al.* Ultrafast carrier dynamics in graphene under a high electric field. Phys. Rev. Lett. 109, 166603 (2012). 23215106 10.1103/PhysRevLett.109.166603

[b9] Graham M. W. *et al.* Photocurrent measurements of supercollision cooling in graphene. Nat. Phys. 9, 103–108 (2013).

[b10] Tielrooij K. J. *et al.* Photoexcitation cascade and multiple hot-carrier generation in graphene. Nat. Phys. 9, 248–252 (2013).

[b11] Tse W.-K. & Das Sarma S. Energy relaxation of hot Dirac fermions in graphene. Phys. Rev. B 79, 235406 (2009).

[b12] Bistritzer R. & MacDonald A. H. Electronic cooling in graphene. Phys. Rev. Lett. 102, 206410 (2009). 19519053 10.1103/PhysRevLett.102.206410

[b13] Xia F. N. *et al.* Ultrafast graphene photodetector. Nat. Nanotechnol. 4, 839–843 (2009). 19893532 10.1038/nnano.2009.292

[b14] Mueller T. *et al.* Graphene photodetectors for high-speed optical communications. Nat. Photonics 4, 297–301 (2010).

[b15] Gan X. *et al.* Chip-integrated ultrafast graphene photodetector with high responsivity. Nat. Photonics 7, 883–887 (2013).

[b16] Pospischil A. *et al.* CMOS-compatible graphene photodetector covering all optical communication bands. Nat. Photonics 7, 892–896 (2013).

[b17] Schall D. *et al.* 50 GBit/s photodetectors based on wafer-scale graphene for integrated silicon photonic communication systems. ACS Photonics 1, 781–784 (2014).

[b18] Boubanga-Tombet S. *et al.* Ultrafast carrier dynamics and terahertz emission in optically pumped graphene at room temperature. Phys. Rev. B 85, 035443 (2012).

[b19] Nair R. R. *et al.* Fine structure constant defines visual transparency of graphene. Science 320, 1308–1308 (2008). 18388259 10.1126/science.1156965

[b20] Shallcross S. *et al.* Quantum interference at the twist boundary in graphene. Phys. Rev. Lett. 101, 056803 (2008). 18764417 10.1103/PhysRevLett.101.056803

[b21] Shallcross S. *et al.* Emergent momentum scale, localization, and van Hove singularities in the graphene twist bilayer. Phys. Rev. B 87, 245403 (2013).

[b22] Shallcross S. *et al.* Electronic structure of turbostratic graphene. Phys. Rev. B 81, 1 (2010).

[b23] Landgraf W. *et al.* Electronic structure of twisted graphene flakes. Phys. Rev. B 87, 075433 (2013).

[b24] Ohta T. *et al.* Evidence for interlayer coupling and moire periodic potentials in twisted bilayer graphene. Phys. Rev. Lett. 109, 186807 (2012). 23215315 10.1103/PhysRevLett.109.186807

[b25] Li G. H. *et al.* Observation of Van Hove singularities in twisted graphene layers. Nat. Phys. 6, 109–113 (2010).

[b26] Yan W. *et al.* Angle-dependent van Hove singularities in a slightly twisted graphene bilayer. Phys. Rev. Lett. 109, 126801 (2012). 23005971 10.1103/PhysRevLett.109.126801

[b27] Havener R. W. *et al.* Angle-resolved Raman imaging of inter layer rotations and interactions in twisted bilayer graphene. Nano Lett. 12, 3162–3167 (2012). 22612855 10.1021/nl301137k

[b28] Ohta T. *et al.* Controlling the electronic structure of bilayer graphene. Science 313, 951–954 (2006). 16917057 10.1126/science.1130681

[b29] McCann E. Asymmetry gap in the electronic band structure of bilayer graphene. Phys. Rev. B 74, 161403 (2006).

[b30] Castro E. V. *et al.* Biased bilayer graphene: semiconductor with a gap tunable by the electric field effect. Phys. Rev. Lett. 99, 216802 (2007). 18233240 10.1103/PhysRevLett.99.216802

[b31] McCann E. & Fal'ko V. I. Landau-level degeneracy and quantum hall effect in a graphite bilayer. Phys. Rev. Lett. 96, 086805 (2006). 16606214 10.1103/PhysRevLett.96.086805

[b32] Guinea F. *et al.* Electronic states and Landau levels in graphene stacks. Phys. Rev. B 73, 245426 (2006).

[b33] Ohta T. *et al.* Interlayer interaction and electronic screening in multilayer graphene investigated with angle-resolved photoemission spectroscopy. Phys. Rev. Lett. 98, 206802 (2007). 17677726 10.1103/PhysRevLett.98.206802

[b34] Zhang Y. B. *et al.* Direct observation of a widely tunable bandgap in bilayer graphene. Nature 459, 820–823 (2009). 19516337 10.1038/nature08105

[b35] Luican A. *et al.* Single-layer behavior and its breakdown in twisted graphene layers. Phys. Rev. Lett. 106, 126802 (2011). 21517338 10.1103/PhysRevLett.106.126802

[b36] dos Santos J. *et al.* Graphene bilayer with a twist: electronic structure. Phys. Rev. Lett. 99, 256802 (2007). 18233543 10.1103/PhysRevLett.99.256802

[b37] Coh S. *et al.* Theory of the Raman spectrum of rotated double-layer graphene. Phys. Rev. B 88, 165431 (2013).

[b38] Brihuega I. *et al.* Unraveling the intrinsic and robust nature of van Hove Singularities in twisted bilayer graphene by scanning tunneling microscopy and theoretical analysis. Phys. Rev. Lett. 109, 196802 (2012). 23215414 10.1103/PhysRevLett.109.196802

[b39] Kim K. *et al.* Raman spectroscopy study of rotated double-layer graphene: misorientation-angle dependence of electronic structure. Phys. Rev. Lett. 108, 246103 (2012). 23004295 10.1103/PhysRevLett.108.246103

[b40] Sato K. *et al.* Zone folding effect in Raman G-band intensity of twisted bilayer graphene. Phys. Rev. B 86, 125414 (2012).

[b41] Carozo V. *et al.* Resonance effects on the Raman spectra of graphene superlattices. Phys. Rev. B 88, 085401 (2013).

[b42] He R. *et al.* Observation of low energy Raman modes in twisted bilayer graphene. Nano Lett. 13, 3594–3601 (2013). 23859121 10.1021/nl4013387

[b43] Ni Z. *et al.* Reduction of Fermi velocity in folded graphene observed by resonance Raman spectroscopy. Phys. Rev. B 77, 235403 (2008).

[b44] Liao L. *et al.* van Hove singularity enhanced photochemical reactivity of twisted bilayer graphene. Nano Lett. 15, 5585–5589 (2015). 26151687 10.1021/acs.nanolett.5b02240

[b45] Wang Y. Y. *et al.* Stacking-dependent optical conductivity of bilayer graphene. ACS Nano 4, 4074–4080 (2010). 20518519 10.1021/nn1004974

[b46] Zhou H. L. *et al.* Chemical vapour deposition growth of large single crystals of monolayer and bilayer graphene. Nat. Commun. 4, 2096 (2013). 23803650 10.1038/ncomms3096

[b47] Yan Z. *et al.* Toward the synthesis of wafer-scale single-crystal graphene on copper foils. ACS Nano 6, 9110–9117 (2012). 22966902 10.1021/nn303352k

[b48] Ma T. *et al.* Repeated growth-etching-regrowth for large-area defect-free single-crystal graphene by chemical vapor deposition. ACS Nano 8, 12806–12813 (2014). 25418823 10.1021/nn506041t

[b49] Ni Z. H. *et al.* G-band Raman double resonance in twisted bilayer graphene: evidence of band splitting and folding. Phys. Rev. B 80, 125404 (2009).

[b50] Giovannetti G. *et al.* Doping graphene with metal contacts. Phys. Rev. Lett. 101, 026803 (2008). 18764212 10.1103/PhysRevLett.101.026803

[b51] Zhou S. Y. *et al.* Substrate-induced bandgap opening in epitaxial graphene. Nat. Mater. 6, 770–775 (2007). 17828279 10.1038/nmat2003

[b52] Xia F. N. *et al.* Photocurrent imaging and efficient photon detection in a graphene transistor. Nano Lett. 9, 1039–1044 (2009). 19203207 10.1021/nl8033812

[b53] Mueller T. *et al.* Role of contacts in graphene transistors: a scanning photocurrent study. Phys. Rev. B 79, 245430 (2009).

[b54] Song J. C. W. *et al.* Hot carrier transport and photocurrent response in graphene. Nano Lett. 11, 4688–4692 (2011). 21936568 10.1021/nl202318u

[b55] Gabor N. M. *et al.* Hot carrier-assisted intrinsic photoresponse in graphene. Science 334, 648–652 (2011). 21979935 10.1126/science.1211384

[b56] Xu X. D. *et al.* Photo-thermoelectric effect at a graphene interface junction. Nano Lett. 10, 562–566 (2010). 20038087 10.1021/nl903451y

[b57] Sun D. *et al.* Ultrafast hot-carrier-dominated photocurrent in graphene. Nat. Nanotechnol. 7, 114–118 (2012). 22245859 10.1038/nnano.2011.243

[b58] Echtermeyer T. J. *et al.* Strong plasmonic enhancement of photovoltage in graphene. Nat. Commun. 2, 458 (2011). 21878912 10.1038/ncomms1464

[b59] Reina A. *et al.* Transferring and identification of single- and few-layer graphene on arbitrary substrates. J. Phys. Chem. C 112, 17741–17744 (2008).

