# Corrigendum: Selectively enhanced photocurrent generation in twisted bilayer graphene with van Hove singularity

**DOI:** 10.1038/ncomms12378

**Published:** 2016-07-19

**Authors:** Jianbo Yin, Huan Wang, Han Peng, Zhenjun Tan, Lei Liao, Li Lin, Xiao Sun, Ai Leen Koh, Yulin Chen, Hailin Peng, Zhongfan Liu

Nature Communications 7: Article numbber:1069910.1038/ncomms10699 (2015); Published: 03 07 2016; Updated: 07 19 2016.

Technical support for this Article was not fully acknowledged. The Acknowledgements should have included the following: We are also grateful to Dr Alexei Barinov for his kind help during our beamtime at the spectramicroscopy beamline of the Elettra synchrotron.

