# Supplementary material for: Selectively enhanced photocurrent generation in twisted bilayer graphene with van Hove singularity
Source: Nat Commun. 2016 Mar 7;7:10699. doi: 10.1038/ncomms10699 (PMC4786639; doi:10.1038/ncomms10699)
Supplement: Supplementary Information — Supplementary Figures 1-6, Supplementary Notes 1-5 and Supplementary References. [file ncomms10699-s1.pdf]

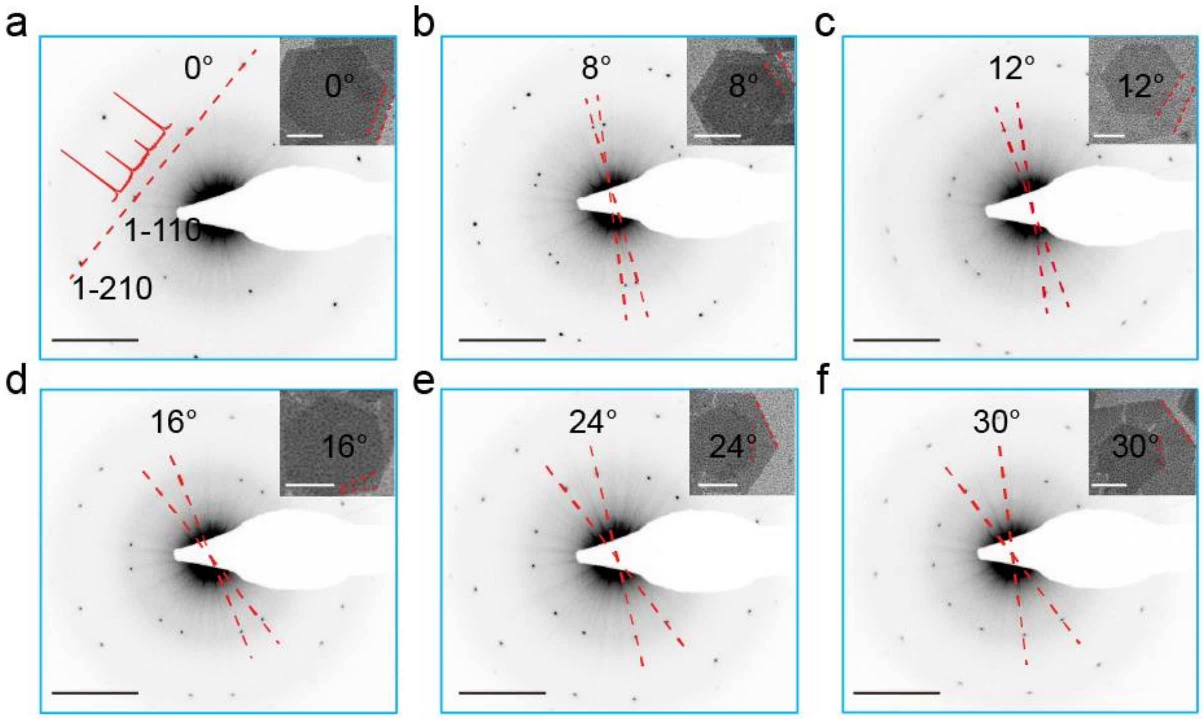

Supplementary Figure 1. Selected area electron diffraction (SAED) of bilayer graphene and tBLG. (a) AB stacked bilayer graphene (b), (c), (d), (e), and (f) are twisted bilayer graphene with twist angle of 8° 12° 16° 24° and 30° respectively.Scale bars: 5 1/nm. Insets are the corresponding SEM images. Scale bars, 5 μm

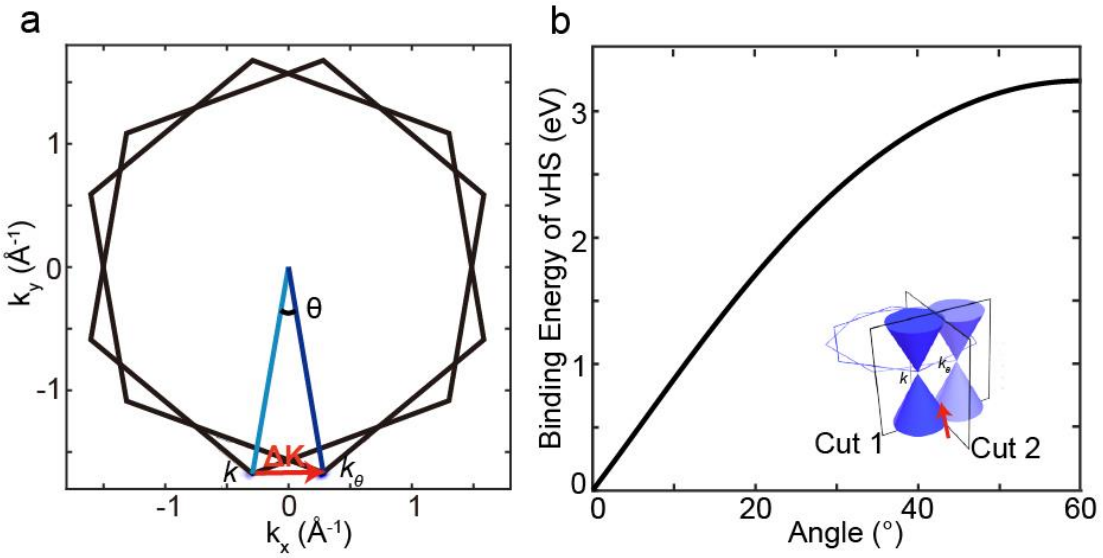

Supplementary Figure 2. Dependence of VHS positions with twited angles. (a) The Brillouin zones of the over- and underlayer of tBLG. (b) The theoretical curve of the binding energy of VHS versus twist angle (θ). Inset is the schematic band structure of tBLG.

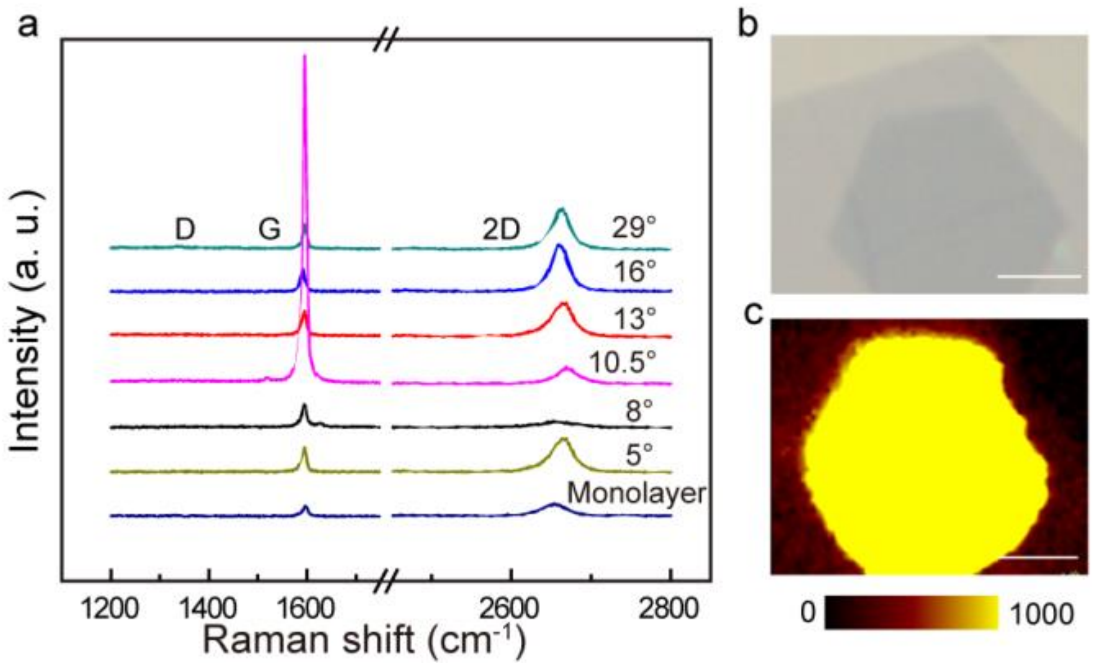

Supplementary Figure 3. Raman spectra of tBLG. (a) Raman spectra of monolayer graphene and tBLG samples with twist angle of 5°, 8°, 10.5°, 13°, 16°, 29° (left column). The incident laser wavelength is 632.8 nm (1.96 eV). The Raman G band of 10.5° tBLG exhibits a ~20 fold enhanced intensity. (b) The optical image of 10.5° tBLG on SiO_2 (90 nm)/Si. (c) The G-band intensity mapping image corresponding to the optical image in (b) shows uniformity of enhancement. Scale bars, 5 μm.

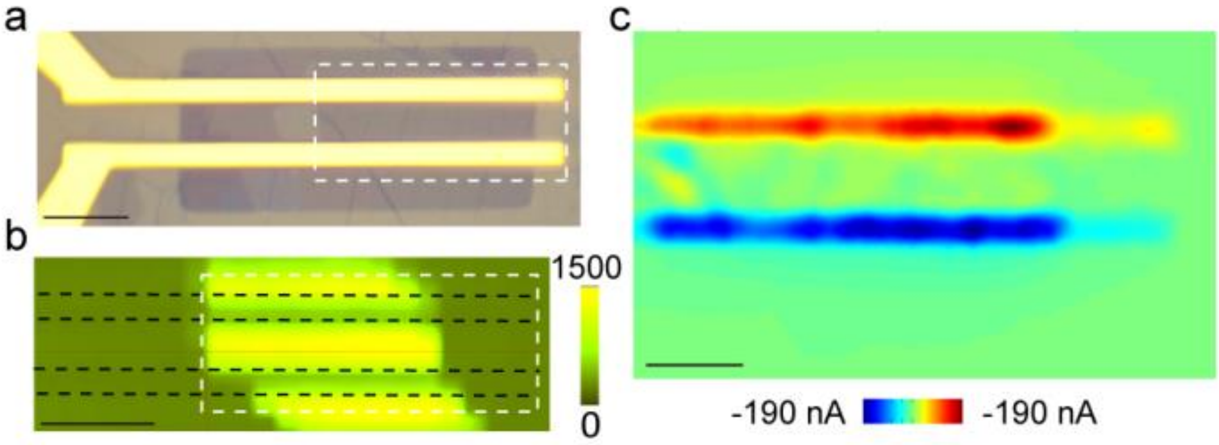

Supplementary Figure 4. Enhanced photocurrent generation in 10.5^o tBLG under illumination of 632.8 nm laser. (a) Optical image of tBLG device. The channel includes 10.5^o tBLG domain. Scale bar 10 μm. (b) Raman G-band mapping image under illumination of 632.8 nm laser (1.96 eV) laser. The bright area indicates the enhanced Raman G-band intensities, which corresponds to 10.5^o tBLG domain. The white dashed rectangle here corresponds to the white dashed rectangle in figure a. The black dashed lines indicate the positions of electrodes. (c) Scanning photocurrent images of the dashed rectangular area of the same tBLG device. A 632.8 nm laser with power of 180 μW is focused on the device, while the net photocurrent is amplified and then detected by a lock-in amplifier. All the photocurrents here are generated without source-drain and gate bias.

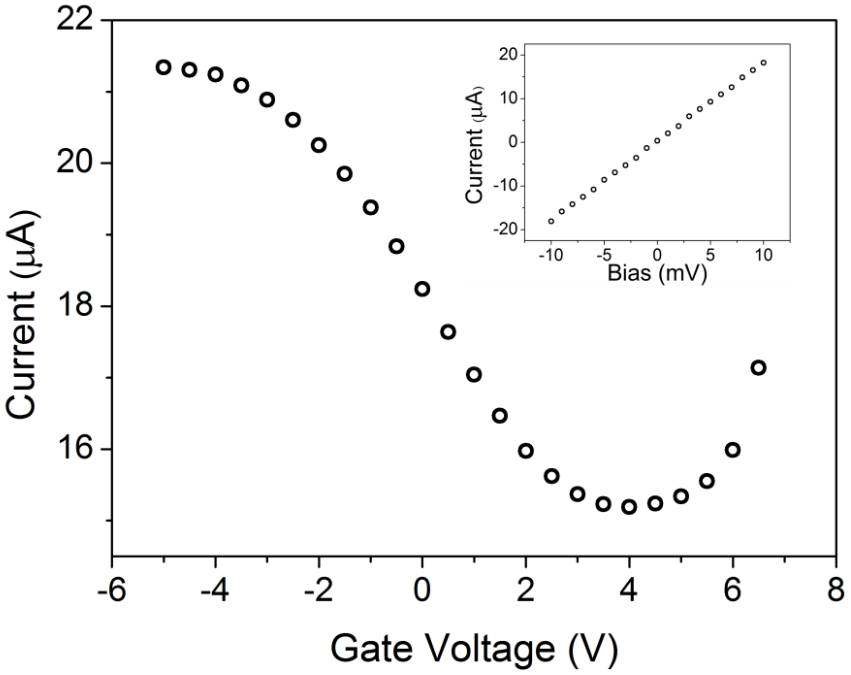

Supplementary Figure 5. The electrical measurement of the tBLG device on silicon substrate with 90 nm SiO_2. The source-drain bias is 10 mV. Inset is the IV curve

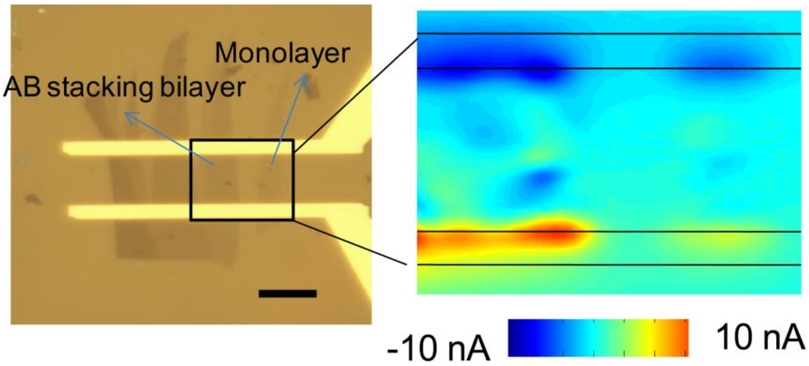

Supplementary Figure 6. Scanning photocurrent study of photodetector based on exfoliated monolayer graphene. (left) Optical image of mechanical exfoliated graphene device. The channel comprises of AB stacking bilayer graphene and monolayer graphene, as shown in black rectangle. Scale bar 10 μm. (right) Scanning photocurrent image of device (labeled by black rectangle in the optical image). The photocurrents were measured under 100 μW 532 nm laser. The black lines indicate the positions of electrodes. All the photocurrents here are generated without source-drain and gate bias.

Supplementary Note 1. The measurement of interlayer twist angle by TEM

The hexagonal shapes of the over- and underlayers of tBLG supply us an opportunity to measure the twist angle (θ) from the linear edges directly. To verify accuracy of this method, we observed the selected area electron diffraction (SAED) of tBLG by transmission electron microscopy (TEM). The tBLG samples with different twist angles were firstly transferred to TEM grids with marks and then identified by scanning electron microscopy (SEM). Finally the SAED of the marked tBLG samples were performed to read the twisted angles from the two sets of diffraction spots which are derived from the over- and underlayers of tBLG. As shown in Supplementary Fig. 1, the twist angles measured from linear edges of tBLG and SAED agree well with each other, but the former method is more convenient. The former method was used to measure the twist angles of tBLG in this work.

Supplementary Note 2. Dependence of VHS positions with twited angles.

Since the over- and underlayer of tBLG are twisted with a rotation angle in real space, their Brillouin zones rotates accordingly (hexagons in Supplementary Fig 2a). The Dirac cones of the over- and underlayer graphene located at $\mathbf{k}$ and $\mathbf{k}_\theta$ points are shown in Supplementary Fig 2a. We define a vector $\Delta\mathbf{k} = \mathbf{k} - \mathbf{k}_{\boldsymbol{\theta}}$, which is the separation between $\mathbf{k}$ and $\mathbf{k}_\theta$ points, then

$$|\Delta\mathbf{k}| = \mathbf{2}|\mathbf{k}|\,\mathbf{sin}\,(\boldsymbol{\theta}/\mathbf{2}) \quad (1)$$

where $|\mathbf{k}| = |\mathbf{k}_{\boldsymbol{\theta}}| = 1.7\text{Å}^{-1}$. The value of $\Delta\mathbf{k}$ can be measured by micro-ARPES, while the value of twist angle θ can be calculated.

The Dirac cones of over- and underlayer graphene of tBLG keep independent near the Fermi level. The energy band of one monolayer graphene has the form of[1]

$$E_{\pm}(\mathbf{k}) = \pm t\sqrt{3 + f(\mathbf{k})} - t'f(\mathbf{k}) \quad (2)$$

where t and t' is the nearest-neighbor hopping energy and next nearest-neighbor hopping energy respectively. The value of t is about 2.8 eV and the value of t' is between 0.02t and 0.2, for example ~0.1 eV[2,3]. On the other hand, the energy band of another monolayer graphene with a twist angle θ, has the form

$$E_{\pm}(\mathbf{k}) = \pm t\sqrt{3 + f(\mathbf{T}(\theta)\mathbf{k})} - t'f(\mathbf{T}(\theta)\mathbf{k}) \quad (3)$$

where T(θ) is the rotation matrix.

If we consider the energy band below the Dirac point, the intersection points are confined by both of the two equations, as

$$\begin{cases} E_1(\mathbf{k}) = -t\sqrt{3 + f(\mathbf{k})} - t'f(\mathbf{k}) \\ E_2(\mathbf{k}) = -t\sqrt{3 + f(\mathbf{T}(\theta)\mathbf{k})} - t'f(\mathbf{T}(\theta)\mathbf{k}) \end{cases} \quad (4)$$

The VHS located around the intersection point with maximum energy value[4,5], while it is the lowest point along the direction perpendicular to Cut 2. As indicated by the red arrow in inset of Supplementary Fig. 2b, it's a saddle points. The relation of this point with twist angle (θ) is derived from the numerical solutions, as shown in Supplementary Fig. 2b.

Supplementary Note 3. Raman G-band enhancement at 10.5^o tBLG domain

The difference between Supplementary Fig. 3a with Fig. 1f is the wavelength of incident laser. Under 532 nm illumination, the tBLG domain with twist angle (θ) of 13° shows a prominent enhanced Raman G-band intensity. However, under 632.8 nm (1.96 eV) illumination, the 10.5^o instead of 13° tBLG domains shows an enhanced Raman G-band intensity. The optical image of 10.5^o tBLG domain is shown in Supplementary Fig. 3b. The Raman G-band intensity mapping of the 10.5^o tBLG domain exhibits a quite uniform feature, which implies a highly crystalline quality (Supplementary Fig. 3c). Although the mechanism of the Raman G-band enhancement is still under controversy, its correlation with the new band topology and VHSs is widely accepted[5-11]. The Raman G band enhancement could be understood by the θ-dependent value of $2E_{\mathrm{VHS}}$, which is defined as the energy interval of the two VHSs (above and under the Dirac point, as shown in Fig. 1a). If this value matches the energy of incident photon, the intensity of Raman G band increases by ~20 folds[5-11]. According to the micro-ARPES data in Fig. 2e, the values of $2E_{\mathrm{VHS}}$ of 13° and 10.5^o tBLG domains are 2.34 eV and 1.89 eV, respectively, which correspond to 2.33 eV (532 nm) and 1.96 eV (632.8 nm). Thus 13° and 10.5^o tBLG domains show enhanced Raman G-band features under illumination of 532 nm and 632.8 nm respectively. Moreover, a mismatch of $2E_{\mathrm{VHS}}$ with $\hbar\omega$ does not lead to the enhancement of Raman G-band intensity. For example, under 532 nm laser illumination, the G-band intensity of tBLG with a twist angle lower or higher than 13° shows a slightly increased or normal value[8] of monolayer graphene, instead of an prominent enhanced value (Fig. 1f), which implies the value of $2E_{\mathrm{VHS}}$ is θ-dependent and different twist angles lead to different values of $2\ E_{\mathrm{VHS}}$. In addition, if the value of $\hbar\omega$ decreases from 2.33 eV (532 nm) to 1.96 eV (632.8 nm), 10.5^o tBLG domain, instead of the 13° tBLG domain shows an enhanced Raman G-band intensity, which implies its value of $2\ E_{\mathrm{VHS}}$ decreases to ~1.96 eV.

Supplementary Note 4. Selectively enhanced photocurrent generation in 10.5^{o} tBLG domain

The tBLG device comprises of tBLG domains with different twist angles (Supplementary Fig. 4a). The enhancement of Raman G-band intensities (Supplementary Fig. 4b) indicates that the tBLG domain has a twist angle of ~10.5^{o}. The energy interval of the two VHSs ($2E_{VHS}$) of 10.5^{o} tBLG domain is approximately 1.89 eV, which matches the energy of incident photon (λ = 632.8 nm, 1.96 eV). As a result, the light-matter interaction is enhanced. The photocurrent was found to be selectively enhanced in a 10.5^{o} tBLG domain device.

Supplementary Note 5. The electrical measurement of the tBLG device on silicon substrate with 90 nm SiO_2.

So far, the reported mechanism of photocurrent generation at the metal-graphene interface is quite controversial. In this article, photothermoelectric (PTE) effect[12-15] was considered to explain the photocurrent generation, but the photovoltaic (PV) effect[16-19] cannot be excluded. In graphene devices, the metal would dope graphene underneath (The doped area was reported to extend into the graphene channel by ~100 nm[16,17]) and then introduce pn junction between graphene underneath and graphene in channel (For some occasions pn junction can also be introduced by the same doping type but with different doping level). From the view of photothermoelectric effect, different values of Seebeck coefficient at the different sides of pn junction introduce anisotropic thermal current flowing. After photoexcitation, a non-zero net PTE current can be generated and detected in the circuit.

The Fermi level of tBLG under the metal is independent on gate voltage and solely controlled by the metal. However the Fermi level of graphene in channel could be tuned by gate voltage. A large positive voltage induces n-type doping to graphene in channel and increases its Fermi level relatively to its Dirac point (The Fermi levels of the graphene sheets under electrodes and in the channel are at the same level if the source-drain bias is zero). As the Fermi level of p-doped graphene increases and passes over the Dirac point, the source-drain current first decreases and then increases as shown at Supplementary Fig. 5. In other words, the doping level of graphene in channel will change relatively to the graphene under the electrode when applying gate voltage. Therefore, the Seebeck coefficient of graphene in channel also changes relatively to the graphene under metal, which may introduce the flips of photocurrent polarity.

Supplementary References

1 Wallace, P. R. The band theory of graphite. *Physical Review* **71**, 622-634 (1947).

2 Deacon, R., Chuang, K.-C., Nicholas, R., Novoselov, K. & Geim, A. Cyclotron resonance study of the electron and hole velocity in graphene monolayers. *Physical Review B* **76**, 081406 (2007).

3 Reich, S., Maultzsch, J., Thomsen, C. & Ordejon, P. Tight-binding description of graphene. *Physical Review B* **66**, 035412 (2002).

4 Havener, R. W., Liang, Y. F., Brown, L., Yang, L. & Park, J. Van Hove Singularities and Excitonic Effects in the Optical Conductivity of Twisted Bilayer Graphene. *Nano Letters* **14**, 3353-3357, (2014).

5 Havener, R. W., Zhuang, H. L., Brown, L., Hennig, R. G. & Park, J. Angle-Resolved Raman Imaging of Inter layer Rotations and Interactions in Twisted Bilayer Graphene. *Nano Letters* **12**, 3162-3167, (2012).

6 Ni, Z. H. *et al.* G-band Raman double resonance in twisted bilayer graphene: Evidence of band splitting and folding. *Physical Review B* **80**, 125404 (2009).

7 Sato, K., Saito, R., Cong, C. X., Yu, T. & Dresselhaus, M. S. Zone folding effect in Raman G-band intensity of twisted bilayer graphene. *Physical Review B* **86**, 125414 (2012).

8 Kim, K. *et al.* Raman Spectroscopy Study of Rotated Double-Layer Graphene: Misorientation-Angle Dependence of Electronic Structure. *Physical Review Letters* **108**, 246103 (2012).

9 Coh, S., Tan, L. Z., Louie, S. G. & Cohen, M. L. Theory of the Raman spectrum of rotated double-layer graphene. *Physical Review B* **88**, 165431 (2013).

10 Carozo, V. *et al.* Resonance effects on the Raman spectra of graphene superlattices. *Physical Review B* **88**, 085401 (2013).

11 He, R. *et al.* Observation of Low Energy Raman Modes in Twisted Bilayer Graphene. *Nano Letters* **13**, 3594-3601, (2013).

12 Xu, X. D., Gabor, N. M., Alden, J. S., van der Zande, A. M. & McEuen, P. L. Photo-Thermoelectric Effect at a Graphene Interface Junction. *Nano Letters* **10**, 562-566, (2010).

13 Gabor, N. M. *et al.* Hot Carrier-Assisted Intrinsic Photoresponse in Graphene. *Science* **334**, 648-652, (2011).

14 Sun, D. *et al.* Ultrafast hot-carrier-dominated photocurrent in graphene. *Nat. Nanotechnol.* **7**, 114-118, (2012).

15 Song, J. C. W., Rudner, M. S., Marcus, C. M. & Levitov, L. S. Hot Carrier Transport and Photocurrent Response in Graphene. *Nano Letters* **11**, 4688-4692, (2011).

16 Xia, F. N. *et al.* Photocurrent Imaging and Efficient Photon Detection in a Graphene Transistor. *Nano Letters* **9**, 1039-1044, (2009).

17 Mueller, T., Xia, F., Freitag, M., Tsang, J. & Avouris, P. Role of contacts in graphene transistors: A scanning photocurrent study. *Physical Review B* **79**, 245430, (2009).

18 Lee, E. J. H., Balasubramanian, K., Weitz, R. T., Burghard, M. & Kern, K. Contact and edge effects in graphene devices. *Nat. Nanotechnol.* **3**, 486-490, (2008).

19 Park, J., Ahn, Y. H. & Ruiz-Vargas, C. Imaging of Photocurrent Generation and Collection in Single-Layer Graphene. *Nano Letters* **9**, 1742-1746, (2009).
